# Connection between academic emotions in situ and retention in the physics track: applying experience sampling method

**DOI:** 10.1186/s40594-018-0126-3

**Published:** 2018-06-20

**Authors:** Sanna Lehtamo, Kalle Juuti, Janna Inkinen, Jari Lavonen

**Affiliations:** 1 Martinlaakson lukio, Vantaa, Finland; 2 Faculty of Educational Sciences, University of Helsinki, Helsinki, Finland; 3 Department of Childhood Education, University of Johannesburg, Johannesburg, South Africa

**Keywords:** Secondary school, Physics, Retention, Emotions, Stress, Experience sampling method

## Abstract

**Background:**

There is a lack of students enrolling in upper secondary school physics courses. In addition, many students discontinue the physics track, causing a lack of applicants for university-level science, technology, engineering and mathematics (STEM) programmes. The aim of this research was to determine if it is possible to find a connection between academic emotions in situ and physics track retention at the end of the first year of upper secondary school using phone-delivered experience sampling method. We applied experience sampling delivered by phone to one group of students in one school. The sample comprised 36 first-year upper secondary school students (median age 16) who enrolled in the last physics course of the first year. Students’ academic emotions during science learning situations were measured using phones three times during each of four physics lessons.

**Results:**

The logistic regression analysis showed that lack of stress predicted retention in the physics track.

**Conclusions:**

Via questionnaires delivered by phone, it is possible to capture students’ academic emotions in situ, information on which may help teachers to support students emotionally during their physics studies. In addition, reflecting their situational academic emotions, students could perhaps make better-informed decisions concerning their studies in STEM subjects.

## Findings

Although students hold the view that science is useful, interesting and important for careers and for society, neither secondary school science nor university-level science is very popular (Osborne and Dillon 2010). Furthermore, too many students leave science programmes that they have entered. The lack of retention of science, technology, engineering and mathematics (STEM) programme is greater during the first year of tertiary level studies (Ulriksen et al. 2010). The lack of engagement in science studies and orientation to science careers has been rather stable, although several projects aiming to make change have been organised. For example, the European Commission’s Horizon 2020 Work Programme (EU 2016) has emphasised that school science should better represent real scientific practices and cater more effectively to the needs and interests of young people. *Inquiry-based science education* (IBSE) has been suggested as a solution to this problem of engaging students in science learning.

The research literature has shown that many factors influence students’ decisions whether to choose science as a course of study or even to discontinue the science track. These factors include science-related ability or self-concept (Nagy et al. 2006; Osborne and Dillon 2010), the perceptions of the future value of science (Osborne and Dillon 2010), students’ positive feelings toward science in an elementary school and middle school before deciding upon the science track at the next school level (Koballa and Glynn 2007), interest in and images of science-related careers (Jones et al. 2000), parental background (Aschbacher et al. 2010; Dustmann 2004), gender (Danielsson 2012; Dweck et al. 2004; Hasse 2002), peer support (Tinto and Cullen 1973) and teacher support (Lengfelder and Heller 2002; Osborne and Dillon 2010; Seymour 2002; Ulriksen et al. 2010).

Based on international comparisons, Ulriksen et al. (2010) concluded that in higher-education mathematics, physics and chemistry, the dropout rate ranged from 31 to 36%, while in pharmacy, biology and geography, the dropout rate was from 6 to 15% (Brown et al. 2008; Nagy et al. 2006; Trumper 2006). However, the first year in any learning institution seems to be the critical one, when decisions to stay or leave are most often made (Tinto 2006).

The way in which the science courses are organized, which topics they introduce and what contexts are used do not necessarily support study retention in the course or the study programme. Based on their literature review, Ulriksen et al. (2010) emphasised that the problem could be more institutional than individual. A student’s history of science experiences follows him or her when he or she tries to enter into the culture of a new study programme. Moreover, most of the research on student engagement in science studies and science careers have been conducted through large-scale surveys and interviews. This type of approaches has limitations because they obtain retrospective measures of students’ reports on engagement experiences and their subjective feelings about them (c.f. Schneider et al. 2016). Therefore, it is important to focus on the emotions and engagement of first-year students in situations.

Lengfelder and Heller (2002) interviewed 8 female and 15 male students who had chosen a science track in upper secondary school to better understand why some students like to work hard and develop their skills in the science track. The students mentioned their social background (e.g. the influence of parents and peers), personal goals (e.g. interest, curiosity, enjoyment of the subject) and personal motives as factors that led them to work harder. However, the interview did not raise any education context, like science lessons, related reasons for choosing science courses. In addition, Wagner and Ruch (2015) found that among primary and secondary school students, feelings that included love of learning, perseverance, zest, prudence, gratitude and hope were related to school. In particular, enthusiasm was robustly associated with school achievement.

Pekrun et al. (2011) found that students’ emotions were linked to their appraisals of control and values, motivation, use of learning strategies, self-regulation of learning and academic performance. They concluded that emotions, including hope and anxiety, were related to success and failure. Anxiety could arise when individuals lacked control over their actions or when their expectations were high and they were worried that they could fail, for example, an exam. Further, boredom is induced when an activity lacks any incentive value, and hopelessness indicates an individual’s feelings about uncontrolled achievement (Pekrun et al. 2011).

Traditionally, students’ beliefs, values and emotions have been studied using surveys that the students are asked to complete as they recall their science learning experiences, attitudes and feelings. However, according to Wirtz et al. (2003), when evaluating recalled experiences, students overestimate their positive emotions. Therefore, recalled emotions do not necessarily match the emotions as they were experienced during actual learning situations. In this study, we aimed to test which situational academic emotions, measured during the actual learning situation, could indicate the likelihood of student retention in a secondary school physics track. There is very little data about upper secondary school students’ emotions in actual learning situations (Schneider et al. 2016), and such data could both increase understanding of why students discontinue STEM education and help teachers to improve teaching in ways that increase the retention rate in STEM education.

In this study, we aimed (1) to test a phone-delivered instrument for investigating students’ situational academic emotion during the lesson and (2) to identify which situational emotions could be used to predict students’ retention in (or leaving out) upper secondary school physic tracks.

### Academic emotions in situ

In the educational context, dropping out can be divided into two types: voluntary withdrawal and academic dismissal. Academic dismissal is associated with grade performance, but voluntary withdrawal is associated with a lack of congruency between the individual and the intellectual climate and peer group social system of a learning institution (Tinto and Cullen 1973). Students’ retention in learning institutions is a key issue among researchers. Several studies have indicated the influence of family background (e.g. socioeconomic status, parental educational level), individual attributes (e.g. academic ability, race and gender) and secondary-level school experiences (e.g. high academic achievement) on a student’s integration into social communities, including schools (Braxton et al. 2000; Kuh et al. 2008; Tinto 2006). However, there is evidence that those external factors opting out or dropping out of study programmes do not differ from each other significantly with respect to motivation, performance or study-related behaviour (Seymour 2002). Although there is a body of knowledge on dropping out of study programmes, it is still unclear how students’ situational academic emotions predict retention in STEM programmes. We assume that situational emotions could be a key to identifying students who are likely to continue or discontinue their upper secondary school physics studies.

Academic emotions are those related to achievement activities, the knowledge-generating qualities of cognitive tasks or the contents covered by learning material (Pekrun et al. 2017). Pekrun et al. (2009) linked positive emotions (enjoyment, hope and pride) to performance approach goals and negative predictors (anger, shame, boredom and hopelessness) to performance-avoidance goals. According to Pekrun et al. (2009), a lack of controllability and a person’s negative perception of the value of science may be expressed as negative emotions, including anxiety, hopelessness and shame. However, negative emotions do not automatically mean a lack of control. Sometimes, a well-motivated student will put more effort into solving a problem and will be more persistent. For example, anxiety is a feeling that can lead to either students’ engagement or discouragement (Moeller et al. 2015). In these situations, negative emotions could guide an analytical, detailed and rigid thinking process (Pekrun et al. 2009; Pekrun et al. 2002). Positive emotions arise in situations in which a person’s thinking is holistic, creative and flexible (Pekrun et al. 2009). In other words, negative emotions do not automatically mean poor performance. Therefore, in the present study, we took into account the nuances of situational emotions in academic task situations. Anxiety and stress have a dual role for learning. Small amounts of anxiety and stress may increase engagement, but too much of either may cause disengagement (Schneider et al. 2016; Moeller et al. 2015; Salmela-Aro and Upadyaya 2014; Pekrun et al. 2009).

We are interested in students’ emotions in situ. Thus, as a methodological approach, we used the experience sampling method (ESM) (Schneider et al. 2016). In ESM, students’ experiences are asked several times in actual learning situations. Thus, the study applied ESM in lesson situations in which emotions occurred while students worked in the classroom (Scollon et al. 2009). Because emotions fluctuate and because remembering them is difficult (Wirtz et al. 2003), the study assessed students’ situational academic emotions several times. When situational emotions are asked several times, and students’ responses are aggregated as a mean over the time, the effect of occasional momentary characteristics became small. Therefore, we expect that with ESM, students’ responses represent more their long-lasting academic emotions, and they may have influence on students’ decisions.

### Research question

We are interested in which academic emotions measured in situ are connected to physics track retention. Our primary research question is as follows: What situational academic emotions predict physics track retention of upper secondary school students? Further, does gender or course achievement predict physics track retention? We answered the research question by using logistic regression analysis to analyse students’ responses to ESM questionnaires that were distributed several times during upper secondary school physics lessons.

### Method

In Finnish upper secondary schools, students must take at least one physics course.^1^ In addition, a student can choose to take from one to seven optional physics courses. If a student enrols in optional physics courses, he or she participates in the physics track. Of the seven courses in physics, three are offered during the first year. After beginning the physics track, a student can discontinue physics at any time. Students make their study plans 1 year at a time. The second-year study plan is made during the time the third physics course is offered. Likewise, the third-year study plan is made at the end of the second year, at which time fewer students discontinue. Students who have taken the last two physics courses in the third year have completed the upper secondary school physics track.

This study was conducted at the end of the first year of an upper secondary school, during the third physics course. The core subjects of this third physics course are waves, sound and light. During the data collection for the study, students were learning about mirrors and lenses. As prescribed by the Finnish national core curriculum (NCCBE 2015), the lessons contained diverse activities, including lectures, hands-on lab activities, modelling and traditional problem solving.

### Sample

The sample consists of data from 10 female students and 26 male students, age 15–16 years. Students were studying the first year in one physics course in one upper secondary school in Finland. Altogether, there were 37 students, who were volunteers, and the permission procedure followed the requirements of the school and the municipality: permission was obtained from the school authorities, the parents and the students. During the data gathering, one student’s responses were not received due to a technical problem with the phone. The first author was the group’s teacher.

We obtained school-register data about the course’s grades and enrolment. The decision to continue or not was made during the students’ third and final physics course in the year in which the study was conducted. In a Finnish upper secondary school, students’ achievements are assessed by applying a seven-level scale.

### Instruments and procedure

For the present study, we adapted Hidi et al.’s (2004) idea about the duration of emotions. Emotions fluctuate during a lesson. Situational academic emotions could come and go in a short period and could be low, high or neutral. If some situational academic emotions occurred constantly, we assumed that they could influence individuals’ decisions (c.f. Hidi 2001). We hypothesised that the frequent occurrence of certain high (or low) emotions would predict retention in the physics track. We applied data collection methods similar to those used by Schneider et al. (2016). The duration of the data collection was 1 1/2 weeks (four lessons), including 12 answers for one student. For the lessons, students were given a phone with a questionnaire application PACO that is available for android phones (http://www.pacoapp.com). For the application, it is possible to schedule several questionnaires. Once the questionnaires are downloaded to the application, the phone does not need an Internet connection for answering. However, when the students’ answers are collected from the phones, the Internet access is needed because the application delivers answers to the website prepared for the analysis. Their phones beeped to signal a request to respond to the questionnaire. Students were asked to respond to the questionnaire at the beginning, in the middle and at the end of the physics lessons. The 75-min lesson was divided into these three time points before the data collection started. The idea was to cover the whole lesson better compared to only one signal. Students had 15-min time to start answering to the ESM questionnaire after they had received the signal. All of the students received the signal at the same time. The questionnaire took about 2 min to answer, and the physics lessons were halted while the students responded to the questionnaire. The lessons had been planned so that the teacher did not know the exact time when requests to answer the questionnaire occurred. We ensured that the time required to answer the questionnaire was minimal and would not significantly interrupt the learning activities.

The present study focused on situational academic emotions in physics lessons. Students were asked evaluate to what extent they agree with the statements measured using a four-point Likert scale, with response choices ranging from 1 = I don’t agree at all to 4 = I agree completely. We calculated the average of the each emotion.

To analyse the data, we applied logistic regression with a forward stepwise method. We used the following variables in the analysis: gender; course grade; and feelings of success, confidence, activeness, happiness and enjoyment, anxiety, stress, confusion and boredom.

### Results and discussion

Our aim was to determine whether situational academic emotions influence student retention in an upper secondary school physics track. Table 1 shows the descriptive statistics regarding situational academic emotions for both students who discontinued and those who continued in the physics track.

**Table 1:** Descriptive statistics regarding academic emotions

Emotion	Discontinue			Continue		
	*N*	*M*	StD	*N*	*M*	StD
Success	12	2.51	0.55	24	2.81	0.50
Confidence	12	2.51	0.70	24	2.51	0.77
Activeness	12	2.48	0.53	24	2.54	0.55
Happiness	12	2.59	0.79	24	2.65	0.64
Enjoyment	12	2.69	0.76	24	2.76	0.47
Anxiety	12	1.69	0.80	24	1.52	0.71
Stress	12	2.40	1.12	24	1.56	0.72
Confusion	12	2.36	0.76	24	1.90	0.74
Boredom	12	2.29	0.84	24	2.10	0.63

Logistic regression analysis was conducted to predict the physics track retention of 36 students in one upper secondary school physics course using academic emotions, gender and the course grade as predictors. The predictors were added using a forward stepwise method. A test of the full model against a constant-only model was statistically significant (chi-square = 6.40, *p* < .05 with df = 1).

Nagelkerke’s R^2^ of .23 indicated a moderately weak relationship between the predictor and the retention. Overall, prediction success was 75% (41.7% for discontinue and 91.7% for continue). The Wald criterion demonstrated that a feeling of stress was the only predictor that made a significant contribution to prediction (*p* = .02). Other predictors were excluded from the equation. The Exp(B) value of 0.37 indicates that when stress increased by one unit, the odds ratio was 0.37 times as large, and therefore, the student was 2.7 times more likely to discontinue the physics track. We tested the situational emotions that students experienced during four physics lessons and calculated the averages of their emotion scores. We interpret that a high average meant that certain situational emotion occurred frequently and that could influence on students’ decisions.

One reason that students have reported dropping out of STEM studies is low grades (Brown et al. 2008). However, in the present study, grades did not play a role. Among those who discontinued the physics track, there were students who had achieved well, and conversely, among those who continued, there were students who had not achieved well. In addition, several studies have reported gender differences in relation to physics learning (Aschbacher et al. 2010; Dweck et al. 2004). In this study, gender did not predict physics track retention.

### Limitations

Before drawing conclusions, it is important to note the limitations of the present study. First, the participants were students of the first author and the sample size was very small. Therefore, it is not possible to generalize the results to any population. Still, this research provided novel insights into how students’ academic emotions can be captured in situ and what is the role of these emotions might be in students’ STEM enrolment. Research showed that students’ situational emotions are able to be captured with a phone application and it might be possible to predict students’ decisions according to their emotions in situ*.*

Second, reporting situational emotions is subjective. The participants self-reported the intensity of their situational emotions. Positive and negative situational emotions and the descriptions of them depend on the individual characteristics such as cultural background (Scollon et al. 2009). In addition, researchers and respondents may interpret those described situational emotions differently. Furthermore, it is impossible to distinguish whether a student is answering a certain way because of his or her teaching or because of issues in his or her personal life (Scollon et al. 2009). Moreover, it is impossible to be sure how long lasting each measured situational emotion is, as they could be passing emotions or they could reflect long-lasting moods. However, aggregating several times during longer times in actual learning situations, measured emotions diminish the influence of occasional passing emotions*.*

### Conclusions

There has been a long tradition of large-scale surveys, and continent wide developing initiatives such as the European Commission’s Horizon 2020 Work Programme (EU 2016) to increase students’ participation in STEM studies. However, there is still very little information about the influence of emotions in actual learning situations for students’ intention to gravitate or retention in STEM studies. This study aimed to identify which academic emotions measured in situ could explain physics students’ decisions to continue or discontinue an upper secondary school physics track.

One outcome of the present study was to show with a small sample that it is possible to capture in situ students’ academic emotions*.* The ESM method was used to obtain data about situational academic emotions in an actual learning situation, rather than asking respondents to recall such emotions afterward. In this way, we ensured obtaining accurate information about students’ situational academic emotions.

Another outcome is the plausibility of felt situational emotions influencing students’ retention in the physics track. According to Pekrun et al. (2011), emotions could be indicators that provided insight into understanding students’ behaviour. The present study did not show why students felt stress, but stressors identified previously have included, for example, students’ expectations about their career, future life plans, tests, grades, and homework; their expectations about school; pressure from parents regarding the students’ responsibilities; pressure from social life; and a lack of time (de Anda et al. 2000). The reason for the stress could also be a work environment that is characterised by competition as opposed to collaboration and lacks the support of an advisor in science, as Ferreira (2003) noted when observing careers at a university. However, in the present study, lack of stress was the only emotion that predicted retention in the physics track.

Based on interviews, Lengfelder and Heller (2002) emphasised the teacher’s role in retention. Teachers could provide more personal, encouraging feedback during and after the course. If teachers were aware of students’ emotions, they could take into account students’ expectations and their experiences in the science track when planning and teaching courses. Applying phone-delivered questionnaire, physics teachers could focus more on emotions in classroom situations. As pedagogical tool, phone questionnaire could raise students’ self-awareness of their situational emotion and their possible influence on decisions.

Our results indicated that when a student do not feel continuous stress, an individual more probably continue in the physics track. The model predicted retention well: If a student felt only a little stress, he or she was more likely to continue in the physics track. However, if the student felt a lot of stress, he or she would not necessarily leave the physics track.

A teacher may reduce the feeling of stress. For example, Misra and McKean (2000) found that there were strong negative correlations between college students’ time management behaviour and feeling of stress. The teacher could emphasize co-planning of the course with students. Discussions and room for students’ personal goals of the course and personal scheduling of course tasks could be pedagogical implications to reduce the feeling of stress. Warnecke et al. (2011) found that daily listened guided mindfulness practice reduced medical students’ feeling of stress. Perhaps, mindfulness practices could have a positive effect for high school physics students’ feeling of stress as well.

The results of academic emotions are very tentative and need to be confirmed in other contexts and with a larger sample of students. However, the case in this paper showed the potential of phone-delivered questionnaire of situational academic emotions to predict students’ decisions concerning course enrolment.

## Footnotes

1 During a 6-week course, a student takes 16 lessons of 75 min each. There are approximately three lessons per week. In Finland, upper secondary school lasts for 3 years.
